# Live Attenuated Infectious Bronchitis Virus Vaccines in Poultry: Modifying Local Viral Populations Dynamics

**DOI:** 10.3390/ani10112058

**Published:** 2020-11-07

**Authors:** Miguel Guzmán, Héctor Hidalgo

**Affiliations:** Laboratory of Avian Pathology, College of Veterinary and Animal Sciences, Universidad de Chile, Santiago 8820808, Chile; miguzman.vet@gmail.com

**Keywords:** Infectious bronchitis virus, viral populations, IBV vaccines, recombination, strain distribution, poultry, protectotypes

## Abstract

**Simple Summary:**

Infectious bronchitis (IB) is one of the more prevalent diseases in poultry, and it is caused by a virus belonging to the Coronaviridae family, the infectious bronchitis virus (IBV), a *Gammacoronavirus* which is related to the *Betacoronavirus* SARSCov-2 causing COVID-19 in humans. IB is mainly controlled by biosecurity and vaccines, although, it is a very challenging issue because the viral populations are constantly evolving by several factors. One of these factors is the same vaccines used for IB control, this could explain by recombination, reversion to virulence, or by favoring virus serotype selection. Thus, a human role in the change of viral populations can be identified by the IBV vaccine usage, this must be considered to achieve effective IB control.

**Abstract:**

Infectious bronchitis virus (IBV) remains one of the most important diseases impacting poultry today. Its high adaptive capacity, attributable to the high mutation rate associated with its ssRNA(+), is one of its more important features. While biosecurity procedures and barriers have been shown to be preponderant factors in minimizing the impact of infectious bronchitis (IB), the environment and procedures associated with intensive poultry systems greatly influence the viral population dynamics. High-density poultry flocks facilitate recombination between different viruses, and even with live attenuated vaccines, which can change the dominant circulating field strains. Furthermore, the remaining issue of reversion to virulence gives rise to significant problems when vaccinal strains are introduced in places where their pathogenic variants have not been reported. Under specific conditions, live attenuated vaccines could also change the frequency of circulating viruses and enable replacement between different field strains. In summary, under a comprehensive approach, while vaccination is one of the most essential tools for controlling IB, the veterinarians, farmers, and official services role in its usage is central to minimizing alteration in a malleable viral population. Otherwise, vaccination is ultimately counterproductive.

## 1. Infectious Bronchitis Characteristics and Control

Infectious bronchitis (IB) was first described in 1931 in the USA [1]. The disease is caused by the infectious bronchitis virus (IBV), a *Gammacoronavirus* belonging to Nidoviral order with a 27.6-kb IBV genome that is ssRNA (+). The first two-thirds of the genome encodes for 16 nonstructural proteins (nsp), which are encoded into overlapping open reading frames (ORFs) *1a* and *1b*. The last third encodes for four major structural proteins: Spike (S), Membrane (M), Nucleocapsid (N), and Envelope (E). Spike protein is cleaved in two by host proteases. S1 is responsible for cell attachment and induces neutralizing antibodies; S2 anchors the protein to the virion and has fusion peptides [2]. However, a recent in vitro study showed that the S2 portion is determinant to allow the viral replication in Vero cells [3].

The transcription mechanism of *Nidoviral* viruses has been previously characterized and includes the generation of intermediate subgenomic RNA (RNAsg). Transcription Regulating Sequences (TRS) serve as an indication point for the genes that determine the generation of specific RNAsg [4]. This transcription mechanism has been shown to facilitate the exchange of complete genes between different virions, with the TRS serving as a hot spot, though intra-gene recombination is also recognized [5]. Polyproteins 1a and 1ab are generated by a frameshift −1 in the viral RNA. The proteases included in their genome cleave the polyproteins into 16 nonstructural proteins (nsp), the primary function of which is to participate in the viral replication mechanisms. Of those, nsp 14 has been described with a proofreading function and reduced activity [5]. Thereby, IBV mutation reaches rates of 10^4^ subs/site/year [6].

Even though IBV is highly labile to environmental temperature and detergents, IB remains one of the most prevalent diseases in modern poultry systems. High morbidity, but in most cases, low mortalities characterize IB. However, mortalities have been recorded in the presence of secondary pathogens [7] or when the IBV has renal tropism [8]. The main factor behind its high presence in poultry is its easy transmissibility through respiratory secretions, feces and even vectors and fomites [9]. The high variability observed in IBV due to high mutation rates and recombination between different strains impedes control and is another factor in the high prevalence of the disease. For a more comprehensive understanding of IB strains, Valastro et al. [10] have classified IBV into six genotypes and 27 lineages, according to the phylogenetic clustering of the S1 sequences.

IBV belongs to the same family as the viruses that cause SARS [11], MERS [12], and the recently-described COVID-19 [13], which spread widely around the world, causing the largest pandemic in modern history. Since all of these human outbreaks have been proven to be of animal origin [14], understanding *Coronaviridae* dynamics and the veterinarians and farmers role in changing their populations has become one of today’s leading research topics.

There is currently no commercially available treatment for IB. However, it has been published that vitamin C and E supplementation may significantly improve the humoral response to IBV in broilers after a combination of live-attenuated and inactivated vaccines [15]. Moreover, small interfering RNA (siRNA) has been shown to reduce IBV replication in vivo using transgenic chickens, minimizing the initial inflammatory process and improving weight gain [16].

Given the lack of cost-effective treatment for IB, the most critical control strategies are prevention through biosecurity measures and all-in/all-out procedures, together with immunization of chickens and breeders. A recent study analyzed the phylogeographic dynamics between two major poultry producers in Italy. The authors showed that integration within each of the companies, with all the implied biosecurity measures, is an efficient barrier for limiting IBV spread. They argued that the disparate biosecurity levels between the two companies could facilitate virus movement in a single direction [17]. Moreover, it has been proposed that the different biosecurity cultures in surrounding countries may explain differences in strain circulation within their territories [18].

Several considerations make the vaccination process a significant challenge. Depending on the age, bird type, and the purpose of protection, local availability of vaccines, manufacturer’s instructions for use and the veterinarian’s diagnose, the professional must establish the most appropriate tactic for use of live vaccines through a vaccination program. Thus, there are as many vaccination schedules as veterinarians prescribing vaccines according to farms circumstances. The first step to choose the most appropriate vaccine to apply is to identify the field strain responsible for the clinical signs, just so, the veterinarian can adjust its vaccinal schedule in order to control the disease.

There are a wide variety of vaccines, which vary in strains and type of vaccines according to local requirements and legal dispositions (Table 1). The most commonly used vaccines are live attenuated vaccines, which mimic the natural infection process of the field viruses without causing the disease. They are produced through an expensive process using embryonated eggs. While it is true that the production of such live vaccines has been industrialized for decades and nowadays it has been established as a massive production through a flow production, to develop for first-time a new vaccine based on a newly isolated field strain takes a long time.

Beyond virus variability, several technical issues affect the outcome of a successful immune response. When evaluating IgM levels, flock characteristics like type of bird, flock size, housing type, ventilation and light management showed significant importance [19]. Similarly, vaccine management factors, including age/interval of vaccination, the interval between vaccination and blood sampling, and temperature of the water used to reconstitute the vaccine, also affect the IgM levels. Furthermore, a recent study made with the Arkansas serotype has shown that the variability in the subpopulations within an available vaccine could define the response of the bird. A more heterogeneous vaccine appears to inhibit viral replication of pathogenic strains more efficiently and to confer a better innate immune response. On the other hand, higher avidity and antibody levels were observer with a more homogeneous vaccine [20].

## 2. Recombinant Strains Retrieved from Field Cases

Several articles report recombination processes between field viruses (Table 2). Isolates from Taiwan have shown recombination of strains from the USA and China in the *N* gene, which may have been involved in the origin of the strains circulating in Taiwan [21]. In another study involving Taiwanese isolates, the authors found multiple recombination points along the IBV genome associated with genes *S, 3b, M,* and *5a* from two different phylogenetic clusters, referred to as TW-I and TW-II [22] but, more recently, grouped in the GI-7 lineage [10].

In Australia, a geographically isolated country, poultry farmers only use live attenuated vaccines manufactured with their own pathogenic strains. Thus, the exchange of viral strains with other countries is extremely limited, and the viral population evolves separately from the rest of the world. In this context, several recombination points have been identified along the entire IBV genome, where field and vaccine strains have been recognized as parental strains [23]. Another study with these Australian strains indicates that natural recombination between distant local strains could be responsible for the emergence of a new variant. Interestingly, a strain that has been undetected for the last decade appears to be involved as a parent strain, suggesting that it could still be circulating at a lower frequency [24].

The full-length sequence and recombination analysis of the CK/CH/Zhejiang/06/10 strain isolated in 2010 in the Zhejiang province of China showed that it is a recombinant of the GX-YL9 strain isolated in 2007 that acquired *S1, 3a, 3b, and 3c, M, 4b, 4c,* and *5a* genes from the LX4 strain isolated in 1999 [25]. A more recent study, published in 2016, also observed the involvement of LX4 and TW-I strains as parent strains in a recombination process for new isolates obtained from China. Analysis of the sequences revealed different point mutations, suggesting that recombination and mutation are important drivers in IBV evolution [26].

In order to circumvent the limitations regarding to develop new live attenuated vaccines, several countries have decided to use the protectotype concept, which uses two heterologous vaccines to neutralize the disease, producing an additive effect greater than their protectives capabilities measured by separate [27]. Knowledge about protectotype effector mechanisms has not been fully understood yet, but it has been shown that protection of the cilia brought by the protectotype can reduce secondary bacterial infections and severity of the disease [28], while a differentiate immune response has been shown to be stimulated by two serotypes involved in protectotype [29].

While the 4/91-Mass combination is the most effective protectotype to date, the concept is not exclusive to this combination [30]. Supporting this idea, the 4/91 vaccine was found to stimulate higher IgA levels in the upper respiratory tract than the Mass vaccine, while the inverse relation was observed for cell-mediated immunity. Thus, it has been proposed that protectotype effectiveness could be based on an additive effect at different levels of the host immune response [29].

Several reports support the use of protectotype against different strains [30,31,32,33,34,35]. Veterinarians and researchers agree on the effectiveness of this methodology. For example, recombinant viruses from chickens with various clinical signs have been retrieved in Russia, Sudan, Egypt, and China. Studies of Russian and Ukrainian field viruses recognized 4/91 and H120 as parental strains of QX recombinant strains, specifically in the *S1* gene [36]. In QX Sudanese sequences, viruses isolated from chickens vaccinated with 4/91 and H120 vaccines have been shown to have acquired a fragment from ORF1a belonging to 4/91 vaccine and another fragment from ORF1b from a H120 vaccine [37]. A GI-23 virus isolated in Egypt exhibits recombination with 4/91 vaccine in genes *3a, 3b, E,* and *M*; and with H120 vaccine in genes *4b* and *4c* [38]. Finally, a Chinese virus obtained in 2014 showed a recombination breakpoint within the S1 sequence, with the 5′ segment more similar to H120 vaccine and 3′ segment more similar to 4/91 vaccine [39].

In Chile, the introduction of the 4/91 vaccine was valuable for controlling the outbreaks caused by GI-16 strains [32]. Six years after this foreign IB vaccine genotype was introduced in the country, field strains belonging to GI-13 with high similarity to the 4/91 vaccine were identified, suggesting a change in the genetic and antigenic properties of the circulating virus [40]. Moreover, a sequence isolated in 2015 has a *S1* gene of Q1 origin (GI-16) and a *N* gene from the 4/91 vaccine (GI-13) [41].

The introduction of the 4/91 vaccine where its homologous pathogenic variant has not been previously isolated or reported, remains controversial. The main concern is how this act could affect local viral populations. However, once it has been introduced under the protectotype concept, removing it from the vaccination schedules could be contraindicated because heterologous field strains continue circulating, though the protectotype keeps them at a lower frequency. If only one of the vaccines (Mass or 4/91) is maintained for field vaccination, the resulting poor cross-protection would not control the field strain. Consequently, an increase in viral population size, with the respective outbreaks, would be observed [42].

## 3. Drivers for IBV Evolution

Many different strains have been described since the first IBV reports in the 1930s. Certain strains spread more than others, probably reflecting greater ability to keep themselves in poultry populations. Some authors suggest that the major spread of specific strains is a consequence of using live attenuated vaccines in poultry [10], which is supported by the recent findings in Chilean strains, where after the introduction of the 4/91 vaccine, pathogenic strains belonging to the same lineage were retrieved from field cases with high homology [40]. The role of wild birds in spreading certain IBV strains has also been considered [43]. Thus, the strain migration by different mechanisms would be one an important driver in IBV evolution.

The high variability of IBV has led to descriptions of different viral populations within individual birds, where mutation, recombination, and selection are constantly modeling IBV evolution through intra-host conditions [44]. Moreover, different subpopulations have even been described in commercial vaccines [45]. This fact is supported by Oade et al. [46], who have shown that the attenuation process required to obtain a live attenuated vaccine could generate different mutation patterns with low consensus levels between them. This is important because heterogeneous mutation patterns afford live attenuated vaccines different initial points for evolution.

Mutation and recombination as drivers of IBV evolution is supported by Zanaty et al. [47], who found that the IBV-EG/1586CV-2015 strain arose from the Egy/var I and Egy/var II viruses isolated in Egypt, all of which belong to the GI-23 group [10]. Moreover, several mutations in the Receptor Binding Domain could be affecting virus tropism and affinity [47]. In another Egyptian study, the authors have suggested that the field strain CU/1/ 2014 would be a revertant of the vaccine H120, exhibiting that that the reverse of virulence is a current concern regarding to live attenuated vaccines [38].

Recombination has been described along the entire IBV genome; however, recombination rates are highest in genes encoding for proteins Spike, nsp 3, nsp 2, and nsp 16. Those immediately upstream of the *Spike* gene have the highest number of recombination breakpoints [5]. These recombination processes have even enabled crossing of the inter-species barrier, as was the case of Turkey Coronavirus (TCov), where a gallus gallus IBV recombined with a Spike protein of unknown origin from another Gammacoronavirus. Thus, when challenged with this new Spike protein, the turkeys’ naïve immune system allowed TCov to spread quickly [48].

The field strain replacement facilitated by vaccines is a phenomenon deeply reviewed by Martcheva et al. [37]. The effectiveness of the vaccine on different circulating viruses can trigger a change in the dominant population, facilitating the rise of a less fit strain as the dominant strain is controlled. However, other virus factors, like the basic reproduction number (R0) and invasion reproduction number (Ri), affect strain replacement and, thus, must be considered. R0 indicates strain capacity to spread in a susceptible population, while Ri shows the same in a host population where another strain is already present and at equilibrium. The possibility of coinfection and the super-infection capability of specific strains can also impact the outcome of strain replacement, which will vary according to the vaccination rate reached [37]. The factors previously described regarding the immunization process will also affect the outcome of strain replacement [35].

Vaccination with live attenuated vaccines has been shown to define the viral population circulating in a specific poultry population. Thus, a homologous vaccine applied in the field leads to less variability in the viral population and negative selective pressure. On the other hand, a non-homologous vaccine is unable to neutralize other serotypes than itself, which causes higher variability, generates diversity through characteristics intrinsic to the virus and exerts positive selective pressure. Finally, when no vaccine is available, variability is high, but the circulating strains are under negative selective pressure [49]. These findings are concordant with the results published by Mckinley et al. [50], who found that usage of live attenuated vaccines can reduce variability in field viral populations. Thereby, vaccine choice is a determining factor in controlling IBVs in the poultry flocks.

## 4. Conclusions

Given the lack of effective treatment for infectious bronchitis, the best option for poultry farmers and veterinarians is prevention through biosecurity protocols and vaccination schedules aligned with the local reality. There are inactivated, live attenuated, sub-unitary, vectorized, monovalent, or polyvalent vaccines, but not all of them are available or authorized in every country. Moreover, new strains are continually being reported in territories where they have not previously been detected. Thus, establishing a vaccination program that is adequate in terms of strain choice, type of vaccine, periodicity of vaccination, among other issues, is becoming increasingly challenging.

Live attenuated vaccines are utilized worldwide because they stimulate different pathways of the avian immune system. However, their use remains a sensitive subject because of how it could change field pathogenic populations through recombination along the entire genome, reversion to virulence, or enabling strain replacement. The issue is most concerning when introducing a new vaccine strain in countries where the pathogenic variant has not been previously detected because, once introduced, the new vaccine strain could evolve in unpredictable ways.

To minimize the associated problems, veterinarians must ensure proper vaccine application in the field. Moving forward, researchers should work on alternatives that provide effective protection and avoid the collateral problems associated with live attenuated vaccines. Given IBV characteristics, developing highly adaptable vaccine-production platforms or expanding the concept of a universal vaccine is essential.

## Figures and Tables

**Table 1:** Main vaccines available by strain and type.

Strain	Type
Massachusetts	Live attenuated–Live modified–Inactivated
Ma5	Live attenuated–Inactivated
D274	Live attenuated–Inactivated
M41	Live attenuated–Inactivated
H52	Live attenuated–Inactivated
CR88121	Live attenuated–Inactivated
H120	Live attenuated
4/91	Live attenuated
1/96	Live attenuated
GA-98	Live attenuated
Arkansas	Live attenuated
1212B	Live attenuated
Conneticut	Live attenuated
B48	Live attenuated
VicS	Live attenuated
Armidale	Live attenuated
249G	Inactivated
PL84084	Inactivated
Delaware	Live modified

**Table 2:** Evidence of recombination in strains isolated from different countries.

Representative(s) of Recombinant	Parental Strains	Gene(s) Involved	Country of Origin of Recombinant
TW2575/98	TW2296/95 and CU-T2	*N*	Taiwan
2575/98, 2992/02, 3071/03, 3374/05, 3468/07	1171/92, 2296/95, CK/CH/LDL/97I	*S, 3b, M, 5a*	Taiwan
IBV/Ck/Aus/N1/03, IBV/Ck/Aus/N1/08	IBV/Ck/Aus/N1/88, VicS, IBV/Ck/Aus/Armidale	*1ab, S, 3a, 3b, E, M, N, 3′ UTR*	Australia
CK/CH/Zhejiang/06/10	GX-YL9, LX4	*S1, 3a, 3b, 3c, M, 4b, 4c, 5a*	China
RF/03/2010	4/91 vaccine, QX	*S1*	Russia
RF/01/2010, UKR/02/2009	H120, QX, D274	*S1*	Russia, Ukrania
IBV/Ck/Sudan/AR251–15/2014	4/ 91, H120, ITA/90254/2005	*1ab, S*	Sudan
CU/4/2014	90254/2005, 4/91, H120	*3a, 3b, E, M, 4b, 4c*	Egypt
IBV-EG/1586CV-2015	IBV/EG/ CLEVB1/2012, IBV/EG/12120s	*S1*	Egypt
ck/CH/LHLJ/140906	H120, 4/91	*S1*	China
13347SP_15	12101SP_09, 4/91	*N*	Chile
